# Enhanced molecular analyses by combination of the HOPE-technique and laser microdissection

**DOI:** 10.1186/1746-1596-1-2

**Published:** 2006-03-31

**Authors:** Torsten Goldmann, Renate Burgemeister, Ulrich Sauer, Siegfried Loeschke, Dagmar Silvia Lang, Detlev Branscheid, Peter Zabel, Ekkehard Vollmer

**Affiliations:** 1 Clinical and Experimental Pathology, Research Center Borstel, Germany; 2 P.A.L.M. Microlaser Technologies, Bernried, Germany; 3 Department of Thoracic Surgery, Krankenhaus Großhansdorf, Germany; 4 Medical Clinic, Research Center Borstel, Borstel, Germany

## Abstract

As part of an investigation aimed at illuminating the possibilities and limits of the HOPE-fixation and paraffin-embedding technique we here describe a novel procedure which was developed in order to combine the benefits of the HOPE-technique with the capabilities of laser microdissection. The presented procedure avoids the need for amplification of template-RNA thus facilitating reliable and reproducible results. The excellent preservation of nucleic acids, proteins, and morphology in HOPE-fixed, paraffin-embedded tissues enhances the molecular applications available to date with materials acquired by laser microdissection when compared to formalin fixed, paraffin-embedded tissues, thus substantially extending the methodological panel in tissue based research.

## Introduction

Laser microdissection is a valuable tool for analysis of molecular parameters in purified cell populations or even single cells taken out of their native environment within tissues. This technology requires both an acceptable preservation of morphological details which is needed for exact dissection and the preservation of the nucleic acids which are analyzed. Fixation of tissues with formalin results in well preserved morphology but – to a high degree – leads to degradation of nucleic acids which substantially constricts the spectrum of applicable molecular techniques [4]. The novel HOPE-technique with subsequent paraffin-embedding, as an alternative to formalin, has been shown to result in a morphological preservation comparable to formalin-fixed, paraffin-embedded specimens [6]. Moreover, we described procedures which permit successful application of all common molecular techniques such as *in situ *hybridization [1,4,7], immunohistochemistry without antigen retrieval and for formalin-refractory antigens [1,3], PCR [8,11], RT-PCR [6,11], Western blot [9], Northern blot, and transcription microarrays [2] to HOPE-fixed, paraffin-embedded tissues. HOPE-fixed tissues can be used for preparation of tissue microarrays for enhanced high throughput analyses on the molecular level [5,12]. Using the HOPE-technique as its crucial methodological base, *ex vivo *model systems could be established, e.g. for the simulation of early events in human infections and detection of chemotherapy resistances in human cancer [7,13]. In addition to tissues, cell-culture preparations have been prepared utilizing the HOPE-technique, which were then successfully applied to in situ hybridization targeting mRNA or immunocytochemistry with excellent preservation of morphological details [10]. In this study we describe the use of HOPE-fixed, paraffin-embedded tissues for laser microdissection and subsequent molecular analysis of RNA-transcripts by real time RT-PCR. Results are set in relation to those obtained with formalin fixed, paraffin-embedded tissues from the same lesions. This real time RT-PCR was unambiguously performed without any amplification of RNA or total cDNA, which eliminates the need for such hardly controllable steps necessary in established procedures with formalin fixed tissues.

## Materials and methods

### Sample preparation

The specimens used were tumor-bearing or tumor-free materials (at least 5 cm away from the tumor front) from lobectomy or pneumectomy because of lung cancer. For means of comparison, tissue samples from the same organs were conventionally formalin-fixed and paraffin-embedded or treated according to the HOPE-technique.

Fixation of tissues by application of the HOPE-technique was carried out like previously described (6), starting with an incubation of fresh tissue specimens in an aqueous protection-solution (containing a mixture of different amino-acids) overnight at low temperatures of 0–4°C (DCS, Hamburg, Germany). Incubation in acetone followed at 0–4°C. Tissues were then directly embedded with paraffin.

Tissue sections from HOPE- or formalin fixed and paraffin embedded (FFPE) human lung cancer tissue samples were prepared on membrane mounted slides (PALM MembraneSlides, P.A.L.M., Bernried, Germany).

HOPE-sections were deparaffinized either with isopropanol (2 × 10 min at 60°C) or like the normal FFPE-sections with xylene washes (2 × 10 min at room temperature).

All sections were stained with cresyl violet (1% w/v cresyl violet acetate in 100% ethanol; Aldrich #86,098-0) for 1 min at room temperature, washed shortly in 70% and 100% ethanol and subsequently air dried.

### Laser microdissection and pressure catapulting (LMPC)

LMPC was performed using a PALM MicroBeam system with a pulsed UV-A nitrogen laser (337 nm). Using the 10× objective areas of interest were marked, cut and catapulted into the cap of 0.5 ml microfuge tubes with adhesive filling (PALM AdhesiveCaps, P.A.L.M., Bernried, Germany). Smaller areas were pooled to reach average sample sizes of 0.5–1 million square microns. After completed microdissection the remaining part of each tissue section was cut out with a scalpel and collected in regular 0.5 ml microfuge tube (cut out section).

### RNA extraction

Tissue samples were extracted using the RNeasy Micro Kit (Qiagen, Hilden, Germany, #74004) following the manufacturer's instruction manual. Briefly, the cells were dissolved in 350 μl of RLT lysis buffer, treated with DNaseI after the first washing step according to the manual and finally the purified RNA was eluted in 12 μl of RNase free water.

Spectrophotometry was performed for testing concentration and purity of RNA, which revealed well-preserved RNA of high concentration throughout the samples.

Of each RNA sample 1 μl was tested in an Agilent Bioanalyzer 2100 using the RNA 6000 Pico LabChip kit (Agilent, Waldbronn, Germany, #5065-4473) to determine the RNA integrity.

### Reverse transcription and RT-PCR

The synthesis of cDNA was performed using the 1st strand cDNA synthesis kit for RT-PCR (AMV) (Roche, Penzberg, Germany, #1483-188) following the manufacturer's instructions. Briefly, 8.2 μl of RNA solution were reverse transcribed in a final volume of 20 μl using random primers from the kit to ensure efficient transcription also of fragmented or partially degraded RNA.

RT-PCR was performed using primers for the low abundant reference gene human Hypoxanthine phosphoribosyltransferase (huHPRT, GenBank: M31642, pos. 383–411 and pos. 613-591) producing a specific amplicon of 231 bp.

The PCR reaction was done in a LightCycler Instrument using the Fast Start Master Plus SYBR Green system (Roche, Penzberg, Germany) applying the following conditions: 95°C for 10 min; 50 cycles at 95°C for 10 sec, 67°C for 10 sec, 72°C for 10 sec followed by a melting curve analysis from 70° to 99°C in 0.1°C steps to control specificity. The amount of RT-PCR product was automatically assessed by real time fluorescence detection with the LightCycler software package.

## Results

**Figure 1:**
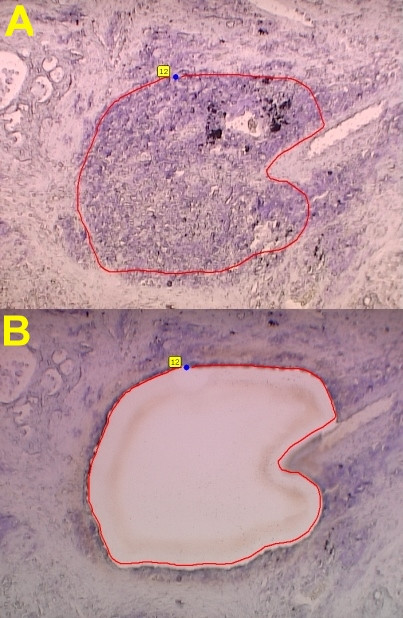
Photomicrographs of HOPE-fixed tissues, deparaffinized and stained with cresyl violet (both 100× magnification). Sections were subjected to LMPC utilizing a PALM MicroBeam system. A: Pulmonary carcinoma with the area subjected for LMPC marked. B: Pulmonary carcinoma with the area subjected for LMPC microdissected and transferred to a microfuge tube by pressure catapulting.

**Figure 2:**
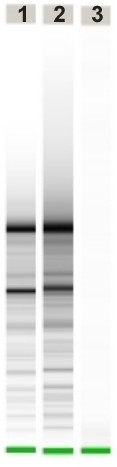
Results of RNA-integrity-testing by a Agilent Bioanalyzer 2100 using the RNA 6000 Pico LabChip kit. Lane 1 and 2 are HOPE-fixed materials and lane 3 is FFPE.

**Figure 3:**
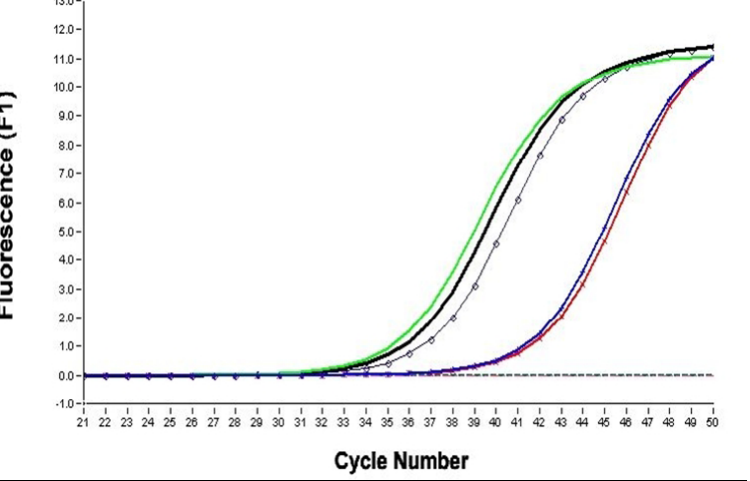
Real time RT-PCR targeting a 231 bp fragment of the RNA of human Hypoxanthine phosphoribosyltransferase. Green: HOPE, deparaffinized with xylene, cut out section. Black drawn through: HOPE, deparaffinized with isopropanol, cut out section. Black with dots: HOPE, isopropanol, whole slice before mounting on slide directly processed. Blue: HOPE, deparaffinized with isopropanol, LMPC. Red: HOPE, deparaffinized with xylene, LMPC. Black noncontinuous: two superposing samples, FFPE, both deparaffinized with xylene, cut out section and LMPC

HOPE-fixed, paraffin-embedded tissues displayed excellent 'formalin-like' preservation of morphological details after sectioning and cresyl violet staining with good adhesion to the membrane mounted slides (PALM MembraneSlides) used for laser microdissection (Fig. 1). Laser microdissection was smoothly possible without any modification to the PALM MicroBeam system; the same holds true for the pressure catapulting of the dissected material into the collection tubes (Fig. 1). Extraction of RNA according to the protocol described above was achieved quickly. RNA-quality of the HOPE-fixed samples analyzed by using the Agilent Bioanalyzer was clearly superior to the formalin-fixed materials, which is exemplified in figure 2. In the real time RT-PCR (Fig. 3) similar results were obtained with deparaffinization utilizing xylene or isopropanol for the samples which have undergone LMPC and the cut out sections. Differences between cut out sections and LMPC can be explained by the differing amounts of starting material. FFPE materials – either LMPC or cut out sections – did not show any amplification of the targeted human Hypoxanthine phosphoribosyltransferase fragment. This is in good agreement with the results of RNA-quality analysis in FFPE materials obtained with the Bioanalyzer, which does not show any visualized RNA in these blocks (Fig. 2).

## Discussion

Laser microdissection for analysis of molecular parameters in cell populations or single cells out of tissues requires acceptable preservation of morphology and nucleic acids. This cannot be achieved to a sufficient degree by the use of FFPE, which display degradation of nucleic acids if compared to fresh materials, which prompted us to this investigation of HOPE-fixed materials.

We showed that HOPE-fixed, paraffin-embedded tissues are well suited for laser microdissection. The well preserved morphology is comparable to formalin-fixed specimens and superior when compared to frozen sections. RNA of high quality can be extracted from the microdissected samples and subsequently being used for successful analysis by real time RT-PCR. These RT-PCR analyses can be performed without the need for any RNA amplification procedure. This results in higher specificity and reproducibility if compared to protocols which have to use such techniques due to degradation within the (usually formalin-fixed) specimens. Therefore results of real time RT-PCR with HOPE-fixed tissues are clearly superior to those obtained in formalin-fixed materials.

The combination of the enhanced molecular possibilities provided by the HOPE-technique with laser microdissection represents a novel tool for future tissue-based studies. Appropriate studies are underway to further extend these promising initial results.
